# An Interplay of Light and Smoke Compounds in Photoblastic Seeds

**DOI:** 10.3390/plants11131773

**Published:** 2022-07-04

**Authors:** Renata Bączek-Kwinta

**Affiliations:** Department of Plant Physiology, Breeding and Seed Science, Faculty of Agriculture and Economics, University of Agriculture in Krakow, Podłużna 3, 30-239 Kraków, Poland; renata.baczek-kwinta@urk.edu.pl

**Keywords:** seed germination, smoke compounds, karrikin, red light, blue light, phytochrome, butenolide, smoke water, mandelonitrile, gibberellin

## Abstract

Light increases the germinability of positively photoblastic seeds and inhibits the germination of negative ones. In an area where plant-generated smoke from fire is a periodically occurring environmental factor, smoke chemicals can affect the germination of seeds, including those that are photoblastically sensitive. Moreover, as smoke and its compounds, mostly karrikin 1, KAR1, have been used for priming the seeds of many species, including photoblastic ones, a systematic review of papers dealing with the phenomenon was conducted. The review indicates that the unification of experimental treatments (light spectrum, intensity and photoperiod, and KAR1 concentration within the species) could improve the quality of global research on the impact of smoke chemicals on photoblastic seeds, also at the molecular level. The review also reveals that the physiologically active concentration of KAR1 varies in different species. Moreover, the physiological window of KAR’s impact on germination can be narrow due to different depths of primary seed dormancy. Another concern is the mode of action of different smoke sources and formulations (aerosol smoke, smoke-saturated water), or pure smoke chemicals. The reason for this concern is the additive or synergetic effect of KARs, cyanohydrins, nitrates and other compounds, and the presence of a germination inhibitor, trimethylbutenolide (TMB) in smoke and its formulations. Obviously, environmental factors that are characteristic of the local environment need to be considered. From a practical perspective, seeds germinating faster in response to smoke chemicals can outcompete other seeds. Hence, a thorough understanding of this phenomenon can be useful in the restoration of plant habitats and the protection of rare species, as well as yielding an improvement in plants that are sown directly to the field. On the other hand, the application of smoke compounds can induce “suicidal germination” in the photoblastic seeds that are buried in the soil and deplete the soil seed bank of the local population of unwanted species.

## 1. Introduction

Seed germination depends on both intrinsic and environmental factors. Among the latter, water, oxygen and temperature are the most important, but for some photoblastic species, light is also crucial, and this is termed photoblastism [1,2,3]. Seed response to light can be positive (germination stimulation) or negative (germination inhibition). While considering the seed size, many small seeds are positively photoblastic, and most pioneering plants produce such seeds [1,2]. Among plant-life form categories, hemicryptophytes and sprouters often produce positively photoblastic seeds, while chamaephytes often produce negatively photoblastic seeds [3]. 

According to the experimentally proven phytochrome response theory, red light (R) is responsible for the germination of photoblastic seeds. This is perceived by a protein-bilin photoreceptor, phytochrome, which also controls blossoming and other physiological responses [4,5,6]. The impact of blue light (B) on germination is considered to be negative [7]. However, the germination of a green vegetable of the Brassicales order, *Cleome gynandra*, is stimulated by blue light [8]. B is perceived by seed cryptochromes and phototropins, and the molecular mechanism of germination under B is under investigation ([9], and the references therein). 

The germination stimulation of photoblastic seeds by smoke was first described in the 1990s. Preliminary studies involved positively photoblastic seeds of ‘Grand Rapid’ lettuce, as well as some Fabaceae and Cistaceae species that were typical of fire-prone environments [10,11,12]. The first substance of physiological activity, 3-methyl-2*H*-furo[2,3-*c*]pyran-2-one, belonging to butenolides, was discovered later and named karrikin 1 (KAR1) [13,14]. However, the existence of smoke-responsive plant species that did not react to KAR1 led to the discovery of other biologically active smoke chemicals: cyanohydrins glyceronitrile (2,3-dihydroxypropanenitrile) and mandelonitrile (MAN), hydroquinones, nitrates, and syringaldehyde [15,16]. These were found to be responsible for germination, and many papers and reviews described the use of some of these compounds as biostimulants [8,14,15,17,18,19,20,21,22]. Moreover, a smoke-derived inhibitor of germination, 3,4,5-trimethylfuran-2(*5H*)-one or trimethylbutenolide (TMB), was identified [17,23,24]. 

The objective of this review is to focus on the research advances in the field of smoke compounds and photoblastism. Examples of species whose seed germination is or can be affected this way, and the practical importance of a technique of smoke-induced germination are provided. The molecular mechanism of action of KAR1 on plant species with photoblastic seeds is summarized. Further, based on the existing data, some questions are also raised. 

## 2. The Impact of Smoke Formulations and Isolated Smoke Compounds on the Germination of Photoblastic Seeds

Table 1 presents examples of different smoke forms and isolated smoke chemicals of physiological importance that are used for treating seeds of different species. The first data linking smoke with its ability to substitute light for positively photoblastic seeds of ‘Grand Rapids’ lettuce come from Drewes et al. [10] and Jäger et al. [11]. They laid the foundation for further research with the use of seed lots of different origin and to check the red/far red (R/FR) seed response to establish the involvement of the phytochrome system in smoke-stimulated seed germination. Merrit et al. [25] provided evidence that KAR1 acts in a similar manner as gibberellic acid when stimulating the germination of some Australian plants. 

Over the last thirty years, the research also included species of different habitats. Do smoke compounds and light act in concert, or can smoke emulate the impact of light? This is difficult to answer based on the literature data. The use of photoperiod for seeds of unknown or undefined photoblastism was probably due to the involvement of natural daylength occurring in the specific area (Table 1). Light source, that is, light spectrum and intensity, varied in different experiments. The seeds were kept under natural light, artificial white light or fluorescent light, which introduced variability among the compared studies. This is discussed in Section 5. While looking for other factors differentiating the experiments on photoblastic seeds, smoke formulations and isolated compounds were noticed. 

When an experimental treatment involves smoke-infused water or smoke fumigation, the question arises as to what should be used as a control. The first attempt toward this was made by Jäger et al. [11]. Aqueous smoke extracts were prepared from a range of plants, and the extracts from agar and cellulose were used. All of them stimulated the germination of lettuce, and a chromatographic analysis indicated the presence of the same compounds, which was a big step towards the discovery of KARs. Today, it is known that the chemical composition of the smoke varies due to specific secondary metabolites and different amounts of various carbohydrates, that is KAR and TMB precursors [26,27,28]. In paper [29], smoke water from two plants, white willow and lemon eucalyptus, was used, and the authors reached the same results in the seeds of different horticultural crops. The author of [30] reported no difference in the effects of smoke that was generated by burning laboratory filter paper or dried meadow sward, which eliminated the potential stimulatory impact of coumarins, secondary metabolites that are abundant in grasses, on germination [31]. In another experiment, [32] the smoke water of an Australian grass, *Themeda triandra* (Poaceae), was used to treat the seeds of a cosmopolitan persistent weed, *Avena fatua*, of Eurasian origin. As reported by Long et al. [33], KAR1 is a smoke-derived compound that is physiologically active toward the seeds of *A. fatua*, so the impact of smoke water on its seeds is independent of the type of plant material that is used for smoke generation [33].

**Table 1:** Germination response of seeds treated with different forms of smoke compounds (chronological order). P—positively; N—negatively photoblastic seeds. Some species respond to light during germination but these are not defined as photoblastic.

Plant Species	Smoke Formulation	Active Substance of Smoke	Light and Illumination Parameters	Mode of Action	Reference
*Lactuca sativa* (Asteraceae, different seed lots) **P**	SW of Australian grass *Themeda triandra* Diluted from 1:2 to 1:10,000	Not specified (mixture of smoke compounds)	Darkness, R 1.8 μmol (quantum) m^−2^ s^−1^, FR 4.5 μmol (quantum) m^−2^ s^−1^, 10-min illumination	1:100, 1:500 and 1:1000 dilutions increased seed germination in darkness, 1:500–1:1000 dilutions substituted for R in the presence of FR in germination stimulation	[10]
*Lactuca sativa* **P**	SW of *Themeda triandra*, *Acacia mearnsii, Eucalyptus grandis, Hypoxis colchicifolia, Pinus patula,* SW diluted from 1:1 to 1:100 on tissue paper.	Not specified (mixture of smoke compounds)	Darkness	Inhibition at 1:1 concentration, stimulation when dilutions of 1:10 or 1:100 were used (depending on the plant source)	[11]
*Angianthus tomentosus, Gnephosis tenuissima, Myriocephalus guerinae, Podolepis canescens, Rhodanthe citrina* (Australian annual Asteraceae)	Butenolide and SW	Butenolide (probably KAR1) and mixture of smoke compounds	570 nm (0.8 μmol (quantum) m^−2^ s^−1^); 640 nm (1.24 μmol (quantum) m^−2^ s^−1^); 720 nm (0.2 μmol (quantum) m^−2^ s^−1^); white light (15.4, μmol (quantum) m^−2^ s^−1^, 1400–700 nm); and continuous darkness.		[25]
*Juniperus procera* (Cupressaceae, Gymnosperms)	SW of *Alnus incana*, a tree belonging to Angiosperms	Not specified (mixture of smoke compounds)	White light/continuous	No effect	[34]
*Arabidopsis thaliana* (Brassicaceae) ecotypes	Karrikin	KAR1	100 μmol (quantum) m^−2^ s^−1^, continuous.For Ler and gal-3 ecotypes, FR 6 μmol (quantum) m^−2^ s^−1^	Stimulation in light (?)	[35]
*Avena fatua* (Poaceae)	SW of Australian grass *Themeda triandra* (Poaceae) Butenolide	Not specified (mixture of smoke compounds) Butenolide (probably KAR1)	8 and 50 μmol (quantum) m^−2^ s^−1^, 16/8 h	Stimulation in darkness	[32]
*Avena fatua, Lolium rigidum, Eragrostis curvula, Phalaris minor, Hordeum glaucum, Ehrharta calycina* and *Bromus diandrus* (Poaceae, Australia)	Karrikinolide	KAR1 (?)	30 μmol (quantum) m^−2^ s^−1^, 12/12 h	Stimulation/inhibition depending on the species	[33]
*Hibbertia* species (Dilleniaceae, Australia)	Karrikin, aerosol smoke	KAR1 (?), mixture of smoke compounds	Fluorescent tubes, 400–700 nm, 50 μmol (quantum) m^−2^ s^−1^, 12/12 h	Stimulation	[36]
*Chrysanthemoides monilifera*, (Asteraceae) Australian weed (two populations)	Karrikins	KAR1 and KAR2		Breakdown of physiological dormancy by KAR1 and germination stimulation in field trials	[20]
*Ansellia africana* (Orchidaceae)	SW, KAR1, TMB	Mixture of smoke compounds, KAR1, TMB	30 μmol (quantum) m^−2^ s^−1^), 16/8 h photoperiod	Stimulation of seed germination and protocorm development by SW, inhibition by TMB	[37]
*Heteropogon contortus* (Poaceae) **P**	Food-grade liquid smoke, SW of xylose, *H. contortus*, KAR1, cyanide, potassium cyanide, MAN (benzaldehyde)	Mixture of smoke compounds, KAR1, benzaldehyde, cyanide, potassium cyanide, benzaldehyde	Daylight supplied by a 60-W incandescent plant light bulb	Stimulation of seed germination by all treatments, with the exception of KAR1	[18]
*Matricaria chamomilla, Solidago* sp., (Asteraceae) **P**	SW of mixture of different European meadow plant species	Not specified (mixture of smoke compounds)	Natural (not specified)	Stimulation in darkness (both species), inhibition in the light (*Solidago*)	[30]
*Bauhinia variegata* (Fabaceae) **N**	SW and KAR1	Mixture of smoke compounds and KAR1	Cool-white fluorescent lamps (approx. 70 µmol (quantum) m^−2^ s^−1^)	Stimulated germination in light conditions	[38]
*Chaenorhinum rubrifolium* (Plantaginaceae)	Smoke, KAR1, MAN, NO and nitrates	Smoke, KAR1, MAN	Daylight (?, approx. 100 µmol (quantum) m^−2^ s^−1^, 12/12 h	Stimulation	[21]
*Heteropogon contortus* (Poaceae) **P**	SW	Not specified (mixture of smoke compounds)	Natural (?), 12/12 h	Stimulation	[39]
*Lactuca sativa* **P**	KAR1, SW and TMB	KAR1, SW and TMB	R (660 nm) and FR(730 nm) for 1 h,	TMB significantlyinhibited germination (33%) in R light Stimulation by KAR1 while FR was used	[28]
*Solidago chinensis,* *Pterocaulon balansae, Stenachaenium megapotamicum* (forbs) *Acanthostyles buniifolius* (shrub) (Asteraceae, South America) *Erianthus angustifolius*, *Aristida laevis* (grasses, South America)	Smoke fumigation	Not specified (mixture of smoke compounds)	White (cool daylight, 300 and 80 lx), 12/12 h	Germination stimulation by low light and smoke	[40]
*Cleome gynandra* (Brassicales), native to Africa **N**	SW and KAR1	Mixture of smoke compounds and KAR1	Fluorescent cool white lamps, 108 W, and R (1.6 μmol (quantum) m^−2^ s^−1^), FR μmol (1.4 quantum) m^−2^ s^−1^), green (0.3 μmol (quantum) m^−2^ s^−1^), blue (0.2 μmol (quantum) m^−2^ s^−1^)	Stimulation by SW in darkness	[8]

Abbreviations: KAR1—karrikin 1; FR—far red; MAN—mandelonitrile; R—red; SW—smoke water.

Experiments employing liquid chromatography/mass spectrometry systems for qualitative and quantitative analysis proved that smoke that was obtained from different plant residues may have different properties [41]. Taking this into account, Gupta et al. [27] proposed a method to standardize the SW composition for seed germination and plant growth stimulation based on a ‘Grand Rapids’ lettuce bioassay, and to estimate the levels of stimulatory (KAR1 and KAR2) and inhibitory (TMB) compounds using a UHPLC-ESI(+)-MS/MS analysis. 

Considering the specificity of the response of positively photoblastic seeds, seven global weeds: *Avena fatua, Lolium rigidum, Eragostis curvula, Phalaris minor, Hordeum glaucum, Ehrharta calycina*, and *Bromus diandrus* to karrikinolide (probably KAR1) seem highly interesting [33]. The germination of *A. fatua* non-dormant seeds was consistently stimulated by KAR in different experiments. On the contrary, the seeds of *L. rigidum* were not stimulated by KAR in any case. A different response was observed in *E. curvula*. Its seeds were unaffected by KAR when freshly collected from the maternal plant, but the dormant ones responded to KAR after cold stratification. All the findings pointed to the conclusion that the response of grasses depended on the species, temperature, presence or absence of light, seed storage history (which implies seed burial and low temperature), and KAR concentration. Another important conclusion was that the so-called window of suitable conditions for responding to KAR was narrow, which is discussed in detail in Section 3. 

**Figure 1:**
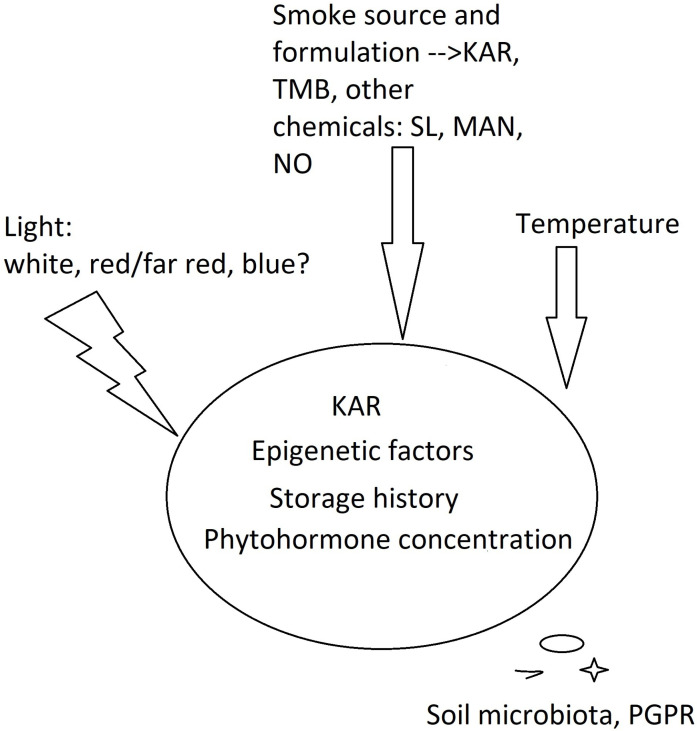
Interaction of KARs, other smoke chemicals and intrinsic and environmental factors on seed germination within the physiological window of KAR perception. PGPR—plant growth-promoting rhizobacteria; MAN—mandelonitriles; NO—nitric oxides; TMB—trimethylbutenolide; SL—strigolactones.

Hidayati et al. [36] revealed a stimulating effect of both KAR1 and aerosol smoke on the seeds of *Hibbertia* sp. (shrubs of Dilleniaceae family), whose germination is very slow (1–2 months for seedling emergence). Some *Hibbertia* plants producing positively photoblastic seeds responded to both treatments, but aerosol smoke provided better results. This means that not only KAR1, but also other smoke-derived compounds stimulated seed germination. In this research and a paper [18], the interest is in the mode of action of different compounds acting individually (KARs, benzaldehydes, cyanohydrins and nitrates) or in concert when aerosol smoke or smoke-saturated water (smoke water, SW) is used. Technically, aerosol smoke and SW contain the same compounds as gaseous products of combustion. However, during storage, various chemical reactions may occur in SW and alter its chemical composition. From a cognitive point of view, such an approach seems interesting, as emphasized already in 2010 by Dayamba et al. [26]. On the other hand, in the research focusing on the cellular mechanism of smoke action in seeds, and in experiments of practical importance, special attention is paid to the impact of substances that are isolated from smoke to obtain reproducibility. This seems justified, as smoke formulations produce different results due to the variable content of germination stimulants and inhibitors [17,23,24]. Following this path of thinking raises another question: do different smoke-derived, physiologically active chemicals act in the same way in photoblastic seeds? To address this query, Tavşanoğlu et al. (2017) [21] studied both KAR1 and a cyanohydrin analogue, mandelonitrile (MAN), in the seeds of an annual plant, *Chaenorhinum rubrifolium*, that was characterized by strong physiological dormancy. Other factors, such as mechanical scarification, heat shock, aqueous smoke, nitrogenous compounds, gibberellic acid (GA), darkness, and photoperiod conditions were also considered. KAR and MAN stimulated the germination of *Ch. rubrifolium,* used both individually and in combination, and the highest germination rate was achieved by a joint treatment with KAR1 and light. Moreover, heat shock and smoke combinations also had positive synergistic and additive effects on germination under light conditions. Therefore, not only the smoke-specific molecules but also environmental factors characteristic of the local environment must be considered (Figure 1).

The additive and/or synergistic effect of the external factors was also investigated by Jurado et al. [42]. They found that environmental features including cold storage, heat, smoke and charcoal (post-fire carbon residue), used individually and in combinations, increased the seed germinability of a Mexican shrub, *Arctostaphylos pungens*. However, the germination rate was relatively low (approx. 30%), even for a combination of cold storage and fire-simulating variables. 

Some cacti produce photoblastic seeds, and it is possible that smoke mimics or modulates the impact of light during their germination [43]. Similarly, Bączek-Kwinta [30] described a stimulating effect of smoke from burning local meadow plants on the positively photoblastic seeds of *Matricaria chamomilla* and *Solidago gigantea* from a European, non-fire prone habitat. The seeds germinated better in darkness after smoke fumigation than in the light. However, the germination of smoke-treated seeds that were exposed to light was delayed. The reason for this could be the generation of reactive oxygen species by some secondary seed metabolites [30].

To summarize, the literature on the stimulation of photoblastic seed germination by smoke-derived compounds shows that the species specificity, smoke formulation, concentration of active smoke chemicals (both stimulants and inhibitors), and environmental factors that are typical of local habitats, as well as the physiological window for seed germination, are important (Figure 1). 

## 3. Physiological Window of KAR1 Perception by Seeds

A limited possibility of KAR perception by seeds was indicated for the first time by Nelson et al. [5]. They proved that KAR1 triggers the germination of the primarily dormant seeds of a model plant species, *Arabidopsis thaliana*, more effectively than the phytohormones of a well-known stimulatory function in germination (GA, EBR (24-epibrassinolide) and ethylene precursor, ACC (1-aminocyclopropane-1-carboxylate)). KAR1 was not equally effective across different *Arabidopsis* ecotypes, which was explained by different depths of the primary seed dormancy. Interestingly, the concentration of ABA, a natural phytohormone keeping seeds in the dormancy state, was unaffected by KAR1. As *Arabidopsis* is a plant that does not grow in fire-prone environments, the authors suggested that KARs belong to the natural plant hormones (or growth regulators) that are involved in seed germination. KARs can also be produced by microorganisms, and a strong selective advantage for species that perceive KARs, such as *Arabidopsis*, is possible [5]. 

Up to now, six KARs have been discovered in plant-derived smoke, and more than 50 analogues have been synthesized [44]. The most often studied natural KAR is KAR1, but the cited research studies revealed the different impact of various KARs on plant seeds [5,20,45]. 

Another interesting fact is that KARs, together with another group of specific biocompounds, strigolactones (SLs), belong to butenolides. Some responses to KARs and SLs can be similar because both groups share common signaling pathways [46,47,48]. This creates new possibilities but also poses new research challenges. 

## 4. Ecological and Practical Implications

The ecological impact of smoke chemicals on photoblastic seeds can sometimes be linked to their practical use, e.g., weed eradication or habitat restoration. Some implications of using smoke formulations or individual smoke-derived compounds, such as KAR1, in fire-prone habitats, are also presented in Table 1. 

The seed germination of *Solidago chilensis, Stenachaenium megapotamicum* and other temperate grassland species of Poaceae and Asteraceae of South American grasslands was affected by smoke and light interaction [40]. The seeds were smoke-fumigated and white light of different intensity simulating the interception of light by a plant canopy was implemented. High light (300 lx) that was associated with smoke treatment inhibited the seed germinability and/or mean germination time, while low light (80 lx) was found to be a key germination cue. The conclusion was that, in such fire-prone ecosystems, fast-germinating seeds responding to low light and smoke compounds after a fire event can outcompete the seeds of other species. 

Considering examples of practical application outside the fire-prone habitats, Bączek-Kwinta [30] postulated the use of SW in the seeds of German chamomile (*Matricaria chamomilla*) that was grown in the field. German chamomile is an important medicinal plant, sown on a large scale in many countries, but its seeds are strongly positively photoblastic. If a heavy rain causes the deep burying of seeds after sowing and negatively affects the yield by diminishing the plant number, the application of smoke compounds could be a solution to the problem. The work of Tavşanoğlu et al. [21] on *Chaenorhinum rubrifolium* (Plantaginaceae) also has an applicative potential, as the studied species is a rare one.

The paper of Leperlier et al. [39] described the impact of smoke-infused water on *Heteropogon contortus,* a savanna plant that is threatened by urbanization, high fire frequency and cattle forage. The seeds of *H. contortus* exhibit a high ability to germinate only between 1 and 2 years after ripening, then the germinability drops. In the cited research, seeds in the first two years after their harvest exhibited physiological dormancy and exposure to light was necessary to enhance their germination. During this period, the SW treatment stimulated the germination of dry stored seeds in both daylight and darkness [39]. The authors emphasize the use of smoke-infused water for large-scale projects of the restoration of savanna, also previously postulated by Baldos et al. [18].

An opposite example of practical use was reported by Flematti [14], who broke the physiological dormancy of an Australian weed, *Chrysanthemoides monilifera*, by using KAR1 (but not KAR2) and stimulating germination in field trials. KAR1 enhanced germination when the seeds were buried in the soil. It seems that KAR1 application can force photoblastic seeds that are hidden in the soil to initiate a “suicidal germination”, resulting in the depletion of the soil seed bank of the unwanted species, and its eradication. 

In the case of negative photoblastism, KAR1 and SW positively affected the germination of the illuminated seeds of a multipurpose tree species, *Bauhinia variegata* [38]. The impact of smoke chemicals on light-induced dormancy was also studied by Nemahunguni [8] on *Cleome gynandra* seeds that failed to germinate when planted immediately after harvest. To enhance better cultivation practices, the seed germination of *C. gynandra* using different light wavelengths (red, far-red, green and blue light) with and without organic biostimulants, including SW and KAR1, was tested. The best germination rate (40%) was observed in the seeds that were treated with SW in the dark. 

Orchids are often negatively photoblastic, and the stimulatory effect of SW in the 16/8 h light regime on germination and protocorm development was reported by Papenfus et al. [37] in leopard orchid, *Ansellia africana*.

In soybean, an important global crop, both light and KAR inhibit germination. The reason for this lies in alterations in the biogenesis ratio of germination stimulants (mostly GA) and germination inhibitor (ABA) [46]. The authors of the paper postulate that spraying the KARs solution on mother plants in the field may decrease pre-harvest sprouting of soybean.

In some cases, smoke and its compounds are not the key cue for germination, even in the fire-prone habitats. In Tigabu et al.’s research [34], an aqueous smoke solution was used to overcome the light that was required for the germination of photo-dormant seeds of *Juniperus procera*, collected from five Ethiopian provenances. The smoke water was obtained by burning samples of *Alnus incana*, a tree belonging to Angiosperms and found in woodlands where *J. procera* grows, so that it emulated a local fire event. However, the treatment was ineffective. In this case, cold-moist stratification (5–10 °C) was the key factor.

Similarly, Ooi et al. [35] examined species that were typical of fire-prone environments in Australia, Africa and South America. Smoke treatment of the seeds of three *Leucopogon* species, whose germination was prevented by the dormancy of a complex character, stimulated germination. Although a long period of darkness or seed burial is necessary to trigger the germination of various *Leucopogon* species, it is hard to establish whether the seeds are negatively photoblastic, and it is more likely that multiple post-fire factors and their interactions are responsible for the germination [35]. This paper confirmed the complexity of the germination of some fire-prone species. In addition, specificity of the soil microbiota can change the mode of action of smoke chemicals [37].

## 5. Disentangling Molecular Mechanism of KAR Action in Photoblastic Seeds

Some of the papers that are cited in this review (Table 1) discussed the impact of R, FR, or B [5,8,28]. In most cases, white light, fluorescent light or scattered daylight was used. As the phytochromes that are involved in seed germination operate at R and FR wavelengths, a differentiated light spectrum could have affected the results. Moreover, the seeds of some species can perceive B [9]. Moreover, day/night light alterations (photoperiod) were in most cases applied according to the natural conditions of a specific geographical region. Future research should be oriented toward unified light conditions (spectrum, and photoperiod) for each photoblastic species.

The question that was raised previously about whether KARs and other smoke-derived compounds can mimic the impact of light in photoblastic species entails the substitution of a phytochrome mode of action. To clarify the role of KAR1 in the photoblastic species, an important piece of work was performed by Gupta et al. (2019) [27]. They studied an antagonistic relationship between KAR1 and a germination inhibitor, TMB, and their physiological impact on the phytochrome-regulated germination of ‘Grand Rapids’ lettuce seeds. The treatment was 1 h of exposure after 3 h of dark incubation, and the control seeds were treated with water. In R light, KAR1 and SW-treated seeds showed a germination rate of 100% and 99%, respectively, while TMB significantly inhibited germination (germination rate 33%). When FR was used, only 6% of the control seeds germinated and the TMB-treated seeds did not germinate at all, while SW and KAR1 significantly reversed the effect of FR; these seeds exhibited a 28% and 35% germination rate, respectively [27]. This indicated that the germination of control lettuce seeds may be modulated by the phytochrome system and/or phytochrome-mediated ABA signaling. According to the paper, a possible mechanism by which seed storage substances are mobilized and germination is achieved may involve the substitution of R and FR with KAR1 and TMB, respectively, via the interconversion of two phytochemical forms of phytochrome, phytochrome red (Pr) and phytochrome far-red (Pfr).

Phytochrome response within plant cells involves gene transcription and/or the expression and activation of epigenetic factors, such as chromatin remodeling and the induction of specific miRNAs. It results in alterations of the phytohormonal balance, whereby ABA level drops and GA and auxin (mostly IAA) levels increase to trigger radicle growth [6], and the references therein. IAA is also involved in regulating seed dormancy [49]. KARs could promote seedling development in *Arabidopsis* and inhibit the expression of *IAA1* [50]. Mutation or a lack of some critical genes within the phytohormonal signaling pathways can also result in a differentiated response mechanism to smoke chemicals.

Blue light should also be considered. As already mentioned, the seeds of *Cleome gynandra* struggle to germinate in white light but germinate better in darkness, which poses a question as to whether light filtering is required for germination [51]. Moreover, Nemahunguni [8] indicated that in this case, R was the factor that inhibited germination, while B stimulated it. A paper on the *Arabidopsis* Columbia ecotype showed that continuous B accelerated the opening of the seed testa and endosperm [52]. The choice of this ecotype was intentional, as it produces particularly fertile and vigorous plants that respond well to changes in photoperiod. The research indicated that phytochromes phyA and phyB, and cryptotropins cry1 and cry2, which are both capable of perceiving B, are required for the blue-light dependent promotion of germination. The study also demonstrated that phyE is important for the first steps of germination in both white and B light [52]. 

**Figure 2:**
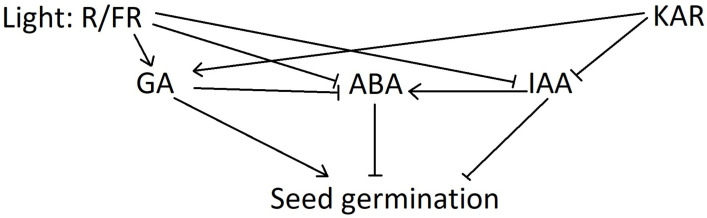
The impact on KAR on phytohormonal balance determining seed dormancy and germination.

Figure 2 depicts the possible mechanism of the interplay between KAR1 and R/FR signaling in the germination of positively photoblastic seeds. In general, germination is under the control of the inhibitors that are involved in seed dormancy (mostly ABA and IAA), while GA stimulates the process. Light, via the phytochrome system, positively affects the GA-inducing *GA3Oxs* gene that is involved in gibberellin biosynthesis. It also decreases the ABA and IAA levels through the inhibition of the *ABA2* and *YUCCA* genes, respectively [53,54,55,56,57,58,59]. Interestingly, KAR1 regulates some light-induced genes such as *GA3Oxs* and *YUCCA* [46]. Currently, the participation of KAR1 in ABA downregulation is not proven [46].

On the other hand, a different light spectrum used in experiments with smoke and/or KAR (Table 1) means the involvement of B, which can modify the light impact on key genes that are involved in germination. In addition, the mechanism definitely differs in some monocots, compared to dicots, because many monocots lack the R/FR response, and W and B light trigger the secondary dormancy of their seeds [57,58]. Moreover, the germination of a dicot, *Cleome gynandra*, is stimulated by blue light [8], and we cannot exclude such a response in other dicotyledonous species. This means that the interplay of smoke-generated volatiles and light can greatly differ within the plant kingdom.

## 6. Conclusions

The impact of smoke-derived compounds on phytohormonal signaling in photoblastic seeds is gradually being revealed. Currently, it is known that KAR1 regulates the light-induced genes that are involved in gibberellin biosynthesis and IAA inhibition. However, the use of different smoke formulations in experiments implies the mode of action of other smoke chemicals. The same refers to different light sources, because the mode of action of R (R/FR), B and W light to seeds varies, especially in dicots and monocots. This review clearly indicates that the impact of smoke chemicals on the germination of crops, model plants and weeds still needs further study at different levels, from the seed biology, through molecular biology and to field trials. Currently, the complexity of seed dormancy and germination is not disentangled; therefore, the photoblastism, as a part of this mechanism, and the impact of KAR1 and other smoke-dependent molecular signals on phytohormonal balance, makes the phenomenon of light-dependent germination worth elucidating.
